# Lung fluid biomarkers for acute respiratory distress syndrome: a systematic review and meta-analysis

**DOI:** 10.1186/s13054-019-2336-6

**Published:** 2019-02-12

**Authors:** Yishan Wang, Huijuan Wang, Chunfang Zhang, Chao Zhang, Huqin Yang, Ruiyue Gao, Zhaohui Tong

**Affiliations:** 1 Department of Respiratory and Critical Care Medicine, Beijing Chao-Yang Hospital, Beijing Institute of Respiratory Medicine, Beijing Engineering Research Center of Respiratory and Critical Care Medicine, Capital Medical University, NO. 8, Gong Ti South Road, Chao-Yang District, Beijing, 100020 China; 2 Department of Anesthesiology, Pain Medicine and Critical Care Medicine, Aviation General Hospital of China Medical University and Beijing Institute of Translational Medicine, Chinese Academy of Sciences, Beijing, 100012 China

**Keywords:** Respiratory distress syndrome, Adult, Acute lung injury, Bronchoalveolar lavage fluid, Biomarkers, Diagnosis, Mortality

## Abstract

**Background:**

With the development of new techniques to easily obtain lower respiratory tract specimens, bronchoalveolar lavage fluid and other lung fluids are gaining importance in pulmonary disease diagnosis. We aimed to review and summarize lung fluid biomarkers associated with acute respiratory distress syndrome diagnosis and mortality.

**Methods:**

After searching PubMed, Embase, Web of Science, and the Cochrane Library for articles published prior to January 11, 2018, we performed a meta-analysis on biomarkers for acute respiratory distress syndrome diagnosis in at-risk patients and those related to disease mortality. From the included studies, we then extracted the mean and standard deviation of the biomarker concentrations measured in the lung fluid, acute respiratory distress syndrome etiologies, sample size, demographic variables, diagnostic criteria, mortality, and protocol for obtaining the lung fluid. The effect size was measured by the ratio of means, which was then synthesized by the inverse-variance method using its natural logarithm form and transformed to obtain a pooled ratio and 95% confidence interval.

**Results:**

In total, 1156 articles were identified, and 49 studies were included. Increases in total phospholipases A2 activity, total protein, albumin, plasminogen activator inhibitor-1, soluble receptor for advanced glycation end products, and platelet activating factor-acetyl choline were most strongly associated with acute respiratory distress syndrome diagnosis. As for biomarkers associated with acute respiratory distress syndrome mortality, interleukin-1β, interleukin-6, interleukin-8, Kerbs von Lungren-6, and plasminogen activator inhibitor-1 were significantly increased in the lung fluid of patients who died. Decreased levels of Club cell protein and matrix metalloproteinases-9 were associated with increased odds for acute respiratory distress syndrome diagnosis, whereas decreased levels of Club cell protein and interleukin-2 were associated with increased odds for acute respiratory distress syndrome mortality.

**Conclusions:**

This meta-analysis provides a ranking system for lung fluid biomarkers, according to their association with diagnosis or mortality of acute respiratory distress syndrome. The performance of biomarkers among studies shown in this article may help to improve acute respiratory distress syndrome diagnosis and outcome prediction.

**Electronic supplementary material:**

The online version of this article (10.1186/s13054-019-2336-6) contains supplementary material, which is available to authorized users.

## Background

Acute respiratory distress syndrome (ARDS) is a clinical syndrome comprising a rapid onset of respiratory failure in patients with risk factors, such as refractory arterial hypoxemia with low reaction to supplemental oxygen and the presence of bilateral infiltrates on radiographic imaging [1]. To date, the diagnosis of ARDS and acute lung injury (ALI) is mostly based on clinical characterization. Frequently-used criteria are the American European Consensus Conference (AECC) criteria [2] and the Berlin definition [3].

As the accuracy of a diagnosis of ARDS based only on the clinical syndrome has been questioned, countless studies have focused on the identification of biomarkers for ARDS. Terpstra et al. conducted a meta-analysis in 2014 focused on plasma biomarkers for ARDS in humans and provided a ranking system for distinguishing the disease from at-risk patients and determining the prognosis [4]. They reviewed multiple plasma biomarkers for ARDS, ranked by pooled odds ratio (OR). However, they only summarized biomarkers for ARDS in plasma; biomarkers in other fluids, such as bronchoalveolar lavage fluid (BALF), were not evaluated.

BALF and other lung fluids, such as pulmonary edema fluid (PEF), epithelial lining fluid (ELF), and lung aspirational fluid (LAF), are definitive in respiratory disease diagnosis. Since BALF provides a sample closest to the site of the disease process, it reflects the local lung environment directly. In 2017, García-Laorden et al. reported that biomarkers representing epithelial apoptosis, such as Fas and FasL, as well as biomarkers reflecting extracellular matrix injury, such as procollagen peptide III (PCP III) and procollagen peptide I (PCP I), were elevated in ARDS BALF samples [5]. The aim of the present study was to compare biomarker levels in lung fluid samples among patients with ARDS and the at-risk controls, as well as those of non-survivors versus survivors of ARDS.

## Methods

### Data source and study selection

We manually searched PubMed, Embase, Wed of Science, and the Cochrane Library for studies on biomarkers for ARDS in lung fluid samples published prior to January 11, 2018. Details of the search strategy are listed in Additional file 1. We also searched the references of included studies. Two researchers screened and evaluated the eligibility of all studies independently, and a third reviewer intervened whenever there was a disagreement. The inclusion criteria were (1) original research report of adult with or at-risk of ARDS, (2) report of exact values of biomarker concentration in lung fluid related to a clinical outcome (diagnosis of ARDS in at-risk patients and/or mortality of ARDS), (3) description of demographic variables, and (4) written in English. The exclusion criteria were (1) written in languages other than English, (2) not related to ARDS/ALI, (3) not an original research, (4) in vivo/in vitro studies, (5) pediatric studies, (6) biomarker not measured in lung fluid, (7) biomarker used for treatment monitoring, and (8) only one article available for a specific biomarker for no mergeable effect size and low reliability.

### Data extraction and quality assessment

We built Excel spreadsheets (Microsoft Corp., Redmond, WA) to extract data from the included studies, and the two researchers finished data extraction independently. The ARDS etiology and the mean or median level and standard deviation (SD) of the biomarker in the lung fluid were obtained. When a biomarker was measured sequentially, only the day 1 measurement was extracted. We extracted lung fluid biomarker levels from different subgroups as follows: patients with ARDS versus critically ill non-ARDS controls and survivors versus non-survivors in patients with ARDS. The mean value of a biomarker’s concentration was equal to the median level in this study, and standard error (SE) was converted to SD using an Excel formula. In addition, demographic variables (age, sex, and number of participants for each subgroup), diagnostic criteria for ARDS, ARDS mortality, the moment the lung fluid sample was retrieved, the sample type (BALF/other than BALF), sample retrieval location, and volume of BALF irrigation solution used were recorded. The recovery rate of BALF was also recorded, if provided.

All studies were assessed for quality according to the Quality Assessment of Diagnostic Accuracy Studies Score-2 (QUADAS-2), and the content was tailored according to the guideline of QUADAS-2 [6]. Details of the tailored QUADAS-2 are listed in Additional file 2. Risk of bias and an applicability concerns graph/summary was conducted using Review Manager version 5.3 (Cochrane Collaboration, Oxford, UK).

### Data synthesis and data analysis

Meta-analysis was performed with Stata 13.1 (StataCorp LLC, College Station, TX). The ratio of means (RoM) was employed to assess the effect size [7–9]. RoM is the mean value of a biomarker in the ARDS group divided by the mean value in the at-risk group (mean_ARDS_/mean_at-risk_) or the mean value of a biomarker in the non-survivors group divided by the mean value in the survivors group (mean_non-survivor_/mean_survivor_). RoM of each study was log transformed and pooled using the inverse-variance method to gain a pooled, transformed RoM, which was then back-transformed to determine the pooled RoM and 95% confidence interval, using the fixed effect model of the Stata software. The significance level for this meta-analysis model was set at *p* < .05. Forest plots were provided for biomarkers of which four or more studies were included in this meta-analysis. Biomarkers were ranked according to pooled RoM and statistical significance. We used the *Q* statistic to test the existence of heterogeneity; a *p* value of less than 0.10 was considered significant for heterogeneity. *I*^2^ was employed to assess the proportion of total variability due to heterogeneity. An *I*^2^ value of approximately 25% was regarded as low heterogeneity, 50% as medium, and 75% as high heterogeneity. Publication bias was assessed with Egger’s regression test [10], where a *p* value of less than 0.10 was considered significant for publication bias. Duval and Tweedie’s trim and fill was then conducted [11].

For the biomarkers with a significant RoM and existence of heterogeneity, we performed a subgroup meta-analysis on study type (case-control study versus another study type) or sample type (BALF versus other lung fluid), when three or more studies were included.

## Results

### Literature search

**Fig. 1:**
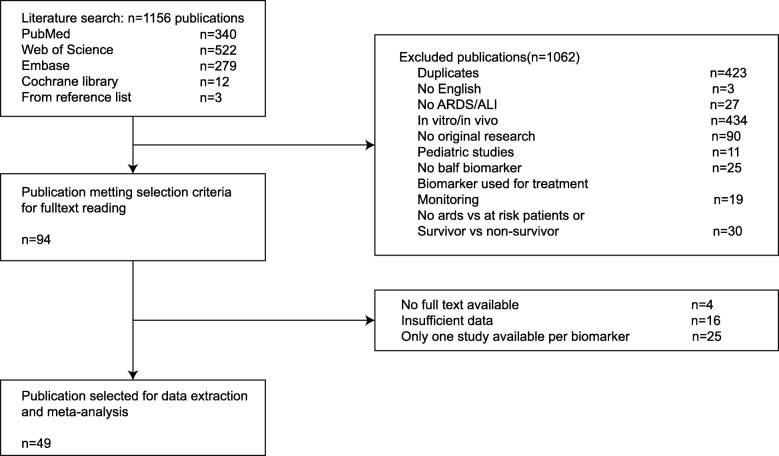
Flowchart of study selection. ARDS, acute respiratory distress syndrome; ALI, acute lung injury; vs, versus

The total literature search yielded 1156 articles from the databases as follows: PubMed, 340 articles; Web of Science, 522 articles; Embase, 279 articles; Cochrane Library, 12 articles; and 3 articles from the reference lists of included studies. By reviewing the titles and abstracts, studies were mainly excluded due to the following: in vitro/animal studies (*n* = 434), duplication (*n* = 423), not an original research (reviews, editorials, or case reports, *n* = 90), and biomarkers not related with occurrence or mortality of ARDS (*n* = 30). After the initial screening, 95 articles remained for full-text review. Of these, 25 articles only reported on a specific biomarker, 16 articles contained insufficient data, and 4 articles had no full-text copy available, despite attempts to contact the authors. The remaining 49 articles were used for the meta-analysis [12–60] (Fig. 1).

### Study characteristics and quality assessment

Demographic variables of the included studies are summarized in (Table 1). A total of 49 articles involving 2189 patients were included in this meta-analysis. ARDS/ALI was diagnosed according to the AECC criteria in 71% of the studies. Other criteria, such as edema fluid/plasma protein ratio [61], lung injury score [62], Fowler criteria [63], and clinical criteria, were used along with the AECC criteria. The mean age ranged from 37 to 70 years, mortality rate ranged from 15 to 77%, and lung fluid was collected between 30 min of intubation and 72 h of ARDS diagnosis. As for sample retrieval location, the right middle lobe and lingular lobe were the most common. Other locations were based on abnormal areas identified on chest radiographs, and a blind sampling of BALF was performed in two studies. In regard to sample type, 69% of the studies measured biomarkers in BALF with a certain volume of irrigation solution. Ten articles used pulmonary edema fluid, and four articles measured a biomarker in epithelial lining fluid. Only three articles provided the recovery rate of the irrigation solution; therefore, we could only assume a stable recovery rate between subgroups for this study.

**Table 1:** Demographic variables

Reference	Biomarkers	Diagnostic criteria for ARDS	Study size	Male (%)	Age	Mortality (%)	Sample retrieved time	Sample retrieved location	Sample type (vol. of irrigation solution)
Studies related to diagnosis									
Armstrong [14]	Procollagen peptide I	AECC	66	66.7	64.65	48.5 (nsp.)	Within 48 h of ICU admission	Right middle lobe	BALF (20 mL*6)
Bersten [15]	Total phospholipids	Clinical criteria	21	np.	np.	np.	Within 7 h of study entry	np.	LAF
Calfee [16]	Soluble intercellular adhesion molecule-1	Clinical criteria	67	59.84	50.63	44.79 (hosp mort)	Within 4 h of intubation	np.	PEF
Chollet-Martin [18]	Interleukin-8	LIS	29	np.	62.12	np.	Within 72 h of ARDS diagosis	np.	ELF
	Tumor necrosis factor-α								
	Interleukin-6								
Conner [20]	Soluble intercellular adhesion molecule-1	Clinical criteria+EF/plasma protein	27	np.	np.	np.	Within 1 h of intubation	np.	PEF
Delclaux [21]	Albumin	Clinical criteria	29	71.43	54	64.29 (nsp.)	np.	np.	BALF (3*50mL)
El Solh [22]	Plasminogen activator inhibitor-1	AECC	51	43.14	36.57	5.88 (hosp mort)	Within 8 h of intubation	Blind BAL	PEF
Prabhakaran [52]	Plasminogen activator inhibitor-1	AECC	51	58	50	47 (hosp mort)	Within 12h of intubation	np.	PEF
Song [56]	Plasminogen activator inhibitor-1	Clinical criteria	33	39	70	15 (28-day mort)	When patients were suspected to have VAP	np.	BALF (20mL*3)
Farjanel [23]	Procollagen peptide III	LIS	61	60.7	49.23	47.5 (hosp mort)	3 days after intubation	A subsegmental of middle lobe	BALF (3*50mL)
	Procollagen peptide I								
	Albumin								
Geerts [25]	Club cell protein	AECC	26	73.1	51	np.	Within 12 h of ARDS diagnosis	Right middle lobe	BALF (50mL*3)
	Total protein								
González-López [26]	Interleukin-8	AECC	22	np.	50.1	31.8 (ICU mort)	np.	np.	BALF (20mL*3)
	Matrix metalloproteinases-9								
	Procollagen peptide III								
	Interleukin-6								
	Vascular endothelial growth factor								
Hallgren [27]	Albumin	Clinical criteria	40	66.67	46.87	25 (nsp.)	np.	Right middle lobe	BALF (3*20mL)
Hamacher [28]	Tumor necrosis factor-α	AECC	56	75	45.41	25.07 (hosp mort)	np.	np.	BALF (nsp.)
	Soluble TNF-α receptors II								
	Transforming growth factor-β1								
Idell [29]	Total protein	Clinical criteria	16	np.	np.	np.	Within 96 h of ARDS diagnosis	Right middle lobe/right lower lobe/lingula	BALF (30mL*5)
Jabaudon [60]	Soluble receptor for advanced glycation end products	AECC	60	55	61	66.67	Within 24 h of disease onset.	np.	LAF
Karagiorga [32]	Total protein	Clinical criteria	31	64.52	41.43	22.58 (nsp.)	Within 72 h of ARDS diagnosis	np.	BALF (20mL*6)
	Platelet activating factor-acetyl choline								
	Total phospholipids								
	Total phospholipases A2 activity								
Keane [33]	Interleukin-8	AECC	9	np.	np.	np.	np.	np.	BALF (nsp.)
	Procollagen peptide III								
	Procollagen peptide I								
Kurdowska [36]	Interleukin-8	LIS	26	np.	np.	41.2 (nsp.)	Upon ICU admission	Right middle lobe	BALF (60mL*3)
Lanchou [37]	Matrix metalloproteinases-9	AECC	21	47.6	54	33.3 (nsp.)	Within 24 h of ARDS diagnosis	Right middle lobe	BALF (20mL*3)
	Matrix metalloproteinases-2								
Martin [44]	Total protein	AECC	69	68	45	56 (nsp.)	Within 72 h of ARDS diagnosis	Right middle lobe/lingula	BALF (30mL*5)
Martin [43]	Soluble receptor for advanced glycation end products	AECC	69	59	53.9	46 (nsp.)	np.	np.	BALF (nsp.)
Medford [45]	Vascular endothelial growth factor	AECC	70	40	59.37	38.57 (28-day mort)	With 72 h of intubation	np.	ELF
Miller [46]	Interleukin-8	AECC	35	np.	48.54	np.	Within 30 min of intubation	np.	PEF
Nakos [49]	Total protein	AECC	21	76.2	61	np.	Within 24 h of intubation	Right middle lobe/lingula.	BALF (20mL*6)
	Albumin								
	Platelet activating factor-acetyl choline								
	Total phospholipids								
Nakos [48]	Total protein	AECC	19	66.67	45	33.33 (nsp.)	Within 12 h of intubation	np.	BALF (20mL*6)
	Albumin								
	Platelet activating factor-acetyl choline								
	Total phospholipids								
Nakos [47]	Platelet activating factor-acetyl choline	AECC	31	29	55.54	32.2 (ICU mort)	Upon ARDS diagnosis	np.	BALF (20mL*6)
	Total phospholipases A2 activity								
Park [51]	Tumor necrosis factor-α	AECC	54	59.1	44.67	20.1 (nsp.)	Within 24 h of ARDS diagnosis	Right middle lobe/lingula	BALF (30mL*5)
	Soluble TNF-α receptors II								
	Interleukin-1β								
	Interleukin-6								
Pugin [53]	Interleukin-8	AECC	31	54.84	48.87	57.65 (nsp.)	np.	blind BAL	PEF
	Matrix metalloproteinases-2								
	Procollagen peptide III								
Ricou [55]	Interleukin-6	Clinical criteria+LIS	24	79.2	50.5	33.3 (nsp.)	Within 24 h of ARDS diagnosis	np.	BALF (nsp.)
Stern [57]	Procollagen peptide III	AECC	25	64	67.08	60 (30-day mort)	Within 72 h of ARDS diagnosis	The abnormal area on the chest radiography	BALF (20mL*6)
	Albumin								
	Hepatic growth factor								
Uchida [58]	Soluble receptor for advanced glycation end products	AECC	33	57.67	43	51.33 (hosp mort)	np.	np.	PEF
Studies related to mortality									
Adamzik [12]	Tumor necrosis actor-α	AECC	47	68.09	44.53	36.17 (30-day mort)	Within 24 h of ICU admission	np.	BALF (40mL*4)
	Total protein								
	Interleukin-6								
	Interleukin-2								
	Interleukin-1β								
	Interleukin-10								
Clark [19]	Procollagen peptide III	AECC	117	64.1	42.78	41 (hosp mort)	Within 72h of ARDS diagosis	Right middle lobe/lingula	BALF (30mL*5)
Frenzel [24]	Interleukin-6	AECC	46	60.9	62	45.7 (28-day mort)	Within 96h of intubation	Right middle lobe/lingula	BALF (20mL*5)
	Interleukin-8								
	Tumor necrosis factor-α								
	Interleukin-1β								
	Interleukin-10								
Kondo [34]	Kerbs von Lungren-6	AECC	32	84.38	70.1	31.3 (hosp mort)	Within 24 h of ARDS diagnosis	Right middle lobe	ELF
Lee [38]	Interleukin-8	AECC	31	51.6	54.5	51.6 (28-day mortality)	Within 48 h of ARDS diagnosis	Right middle lobe/lingular	BALF (30mL*5)
Lin [41]	Interleukin-6	AECC	39	69.2	68	43.6 (hosp mort)	Within 24h of ARDS diagnosis	Right middle lobe/lingula	BALF (20mL*6)
	Interleukin-8								
Nathani [50]	Kerbs von Lungren-6	AECC	42	57.1	60.13	np.	Upon study entry	Middle lobe	BALF (50mL*3)
Ishizaka [30]	Kerbs von Lungren-6	AECC	38	77%	68	32 (hosp mort)	Upon onset of ARDS	Right middle lobe	ELF
Studies related to diagnosis and mortality									
Agouridakis [13]	Interleukin-2	AECC	34	74.42	47.79	27.9 (nsp.)	Within 2h of ICU admission	np.	BALF (nsp.)
Chesnutt [17]	Procollagen peptide III	AECC+EF/plasma protein	44	47.73	56.25	63.64 (hosp mort)	Within 1h of intubation	np.	PEF
Jorens [31]	Club cell protein	Fowler+LIS	35	88.6	55	42.9 (nsp.)	Within 12 h of ARDS diagnosis	Right middle lobe	BALF (50mL*3)
Kropski [35]	Club cell protein	AECC+EF/plasma protein	32	46.9	48	56.5 (nsp.)	Within 24 h of intubation	np.	PEF
Lee [39]	IL-8	Clinical criteria	112	78.6	66.5	77.3 (nsp)	With 24h of ICU admission	The most abnormal area on the chest radiography/right middle lobe/lingula	BALF (20mL*6)
	Tumor necrosis factor-α								
	Interleukin-1β								
	Interleukin-6								
	Interleukin-10								
Lesur [40]	Interleukin-2	AECC	33	36.4	51.52	24.2 (hosp mort)	Within 72h of intubation	Right middle lobe/lingula	BALF (20mL*5)
Marshall [42]	Procollagen peptide III	AECC	60	53.33	51.35	31.67 (nsp.)	Within 24h of ARDS diagnosis	np.	BALF (nsp.)
Quesnel [54]	Interleukin-8	AECC	122	64.8	67	33.6 (28-day mort)	np.	np.	BALF (20mL*6)
	Procollagen peptide I								
	Transforming growth factor-β1								
	Hepatic growth factor								
Ware [59]	Vascular endothelial growth factor	AECC	102	62.8	49	60 (nsp.)	np.	np.	PEF

*ARDS* acute respiratory distress syndrome, *AECC* American-European Consensus Conference, *EF* edema fluid, *LIS* lung injury score, *np* not provided, *nsp* not specific, *mort* mortality, *hosp mort* hospital mortality, *ICU* intensive care unit, *BALF* bronchoalveolar lavage fluid, *LAF* lung aspirational fluid, *ELF* epithelial lining fluid, *PEF* pulmonary edema fluid

The ARDS etiologies are summarized in Additional file 3. The most common cause of ARDS was sepsis (30.87%), followed by pneumonia (23.70%), trauma (10.94%), aspiration (8.53%), transfusion (4.23%), and major surgery (3.47%). Other etiologies included vasculitis, retroperitoneal hematoma-DIC, drug overdose, reperfusion injury, and diabetic ketoacidosis.

The quality assessment is displayed in Additional file 4, including the risk of bias and applicability of studies to the review question.

### Biomarkers associated with ARDS diagnosis

**Fig. 2:**
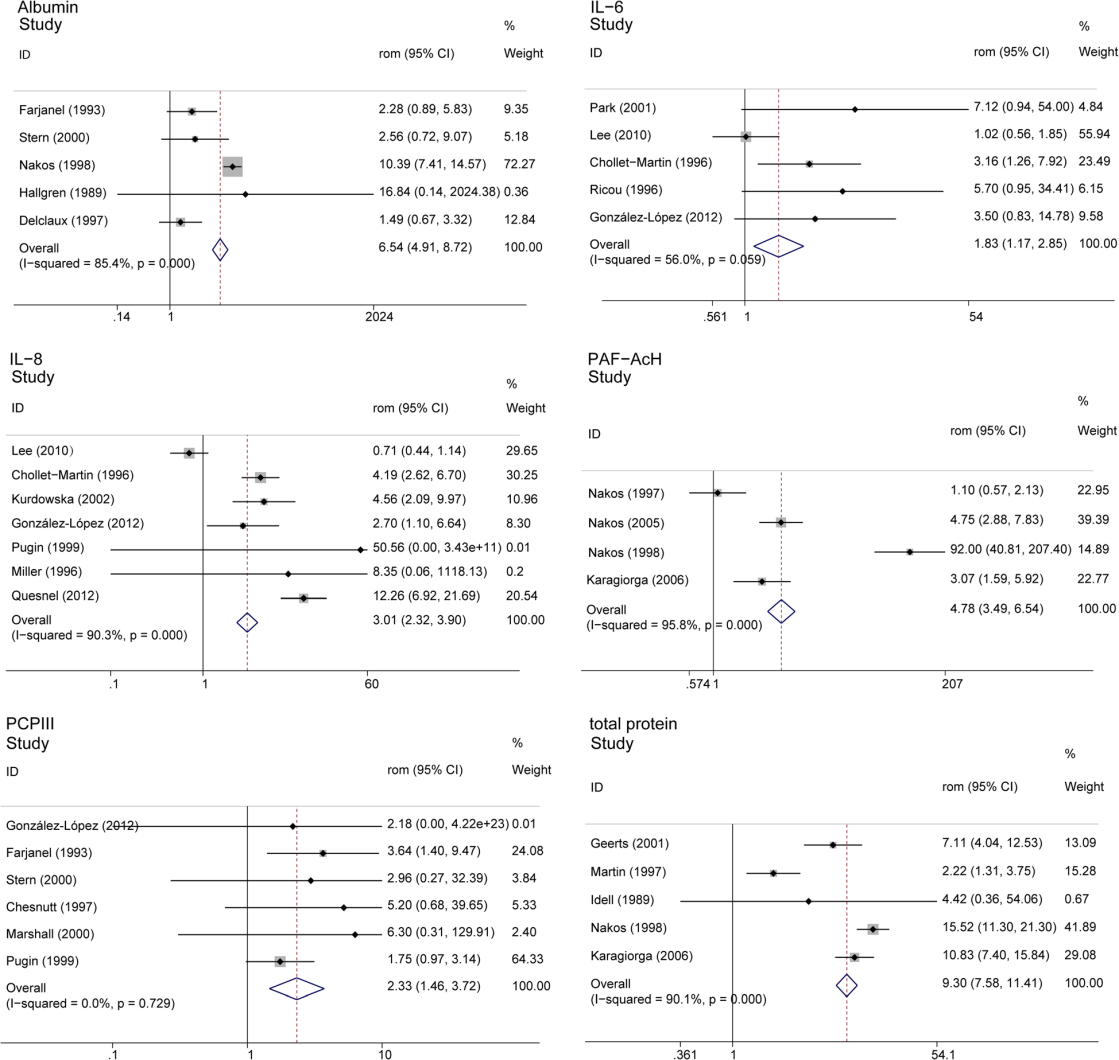
Forest plot for acute respiratory distress syndrome (ARDS) diagnosis. RoM, ratio of means; CI, confident interval; IL-6, interleukin-6; IL-8, interleukin-8; PAF-ACH, platelet activating factor-acetyl choline; PCPIII, procollagen peptide III

We performed a meta-analysis on 22 biomarkers in lung fluid associated with the diagnosis of ARDS in the at-risk population (Table 2); Fig. 2 shows the forest plots for biomarkers available in at least 3 studies. Pooled RoM values for total phospholipases A2 activity (total PLA2 activity) (17.995 [11.381, 28.454]), total protein (9.299 [7.575, 11.414]), albumin (6.544 [4.908, 8.725]), plasminogen activator inhibitor-1 (PAI-1) (5.525 [3.876, 7.877]), soluble receptor for advanced glycation end products (sRAGE) (4.901 [3.603, 7.673]), platelet activating factor-acetyl choline (PAF-AcH) (4.783 [3.495, 6.545]), soluble tumor necrosis factor-α receptors II (STNF-RII) (3.253 [1.765, 5.993]), hepatic growth factor (HGF) (3.199 [1.668, 6.135]), and interleukin-8 (IL-8) (3.008 [2.322, 3.896]) were the highest. The overall effect size ranged from 0.548 to 17.995, among biomarkers with significant RoM between subgroups, and decreased Club cell protein (CC16) (0.553 [0.369, 0.827]) and matrix metalloproteinases-9 (MMP-9) (0.548 [0.336, 0.893]) levels in lung fluid indicated a higher possibility of ARDS diagnosis in the at-risk population. However, a pervasive heterogeneity was displayed.

**Table 2:** Biomarkers associated with ARDS diagnosis

Biomarker	No. of study	No. of patients	RoM (95% CI)	*p*	Heterogeneity	
					*Q* (*p* value)	*I*^2^ (%)
Total phospholipases A2 activity	2[32, 47]	62	17.995 (11.381, 28.454)	< 0.05	10.54 (0.001)	90.50
Total protein	5[25, 29, 32, 44, 48]	179	9.299 (7.575, 11.414)	< 0.05	40.48 (< 0.1)	90.10
Albumin	5[21, 23, 27, 48, 57]	191	6.544 (4.908, 8.725)	< 0.05	27.35 (< 0.1)	85.40
Plasminogen activator inhibitor-1	3[22, 52, 56]	135	5.525 (3.876, 7.877)	< 0.05	3.69 (0.158)	45.8
Soluble receptor for advanced glycation end products	3[43, 58, 60]	162	4.901 (3.603, 7.673)	< 0.05	31.19 (0.000)	93.6
Platelet activating factor-acetyl choline	4[32, 47–49]	120	4.783 (3.495, 6.545)	< 0.05	71.83 (< 0.1)	95.80
Soluble TNF-α receptors II	2[28, 51]	110	3.253 (1.765, 5.993)	< 0.05	4.95 (0.026)	79.80
Hepatic growth factor	2[54, 57]	144	3.199 (1.668, 6.135)	< 0.05	0.02 (0.892)	0
Interleukin-8	7[18, 26, 36, 39, 46, 53, 54]	377	3.008 (2.322, 3.896)	< 0.05	62.08 (< 0.1)	90.30
Soluble intercellular adhesion molecule-1	2[16, 20]	96	2.952 (1.902, 4.581)	< 0.05	0.28 (0.6)	0
Procollagen peptide I	2[14, 23]	133	2.949 (1.867, 4.659)	< 0.05	0.11 (0.743)	0.00
Interleukin-2	2[13, 40]	67	2.761 (1.508, 5.057)	0.001	17.34 (< 0.1)	94.20
Procollagen peptide III	6[17, 23, 26, 42, 53, 57]	195	2.328 (1.456, 3.723)	< 0.05	2.81 (0.729)	0
Interleukin-6	5[18, 26, 39, 51, 55]	250	1.826 (1.170, 2.852)	0.008	9.1 (0.0059)	56
Club cell protein	3[25, 31, 35]	93	0.553 (0.369, 0.827)	0.004	3.6 (0.166)	44.40
Matrix metalloproteinases-9	2[26, 37]	43	0.548 (0.336, 0.893)	0.016	15.45 (< 0.1)	93.50
Transforming growth factor-β1	2[28, 54]	116	1.32 (0.575, 3.034)	0.513	0.93 (0.334)	0
Tumor necrosis factor-α	4[18, 28, 39, 51]	247	1.3 (0.917, 1.843)	0.14	2.97 (0.397)	0
Matrix metalloproteinases −2	2[37, 53]	52	1.066 (0.889, 1.278)	0.493	0.06 (0.814)	0
Total phospholipids	4[15, 32, 48, 49]	110	1.003 (0.862, 1.166)	0.973	34.25 (< 0.1)	91.20
Interleukin-1β	2[39, 51]	166	0.952 (0.628, 1.444)	0.817	4.23 (0.04)	76.30
Vascular endothelial growth factor	3[26, 45, 59]	194	0.812 (0.544, 1.212)	0.309	3.4 (0.183)	41.20

Numbers within the square brackets were reference numbers

*RoM* ratio of means, *CI* confident interval

We performed an influence analysis to examine the sensitivity of the results. Influence analysis showed that the heterogeneity was possibly caused by the limited number of studies. By removing the studies with extreme RoM, we observed a robust effect on the biomarkers. The outcome of the influence analysis is displayed in Additional file 5.

Subgroup analysis was performed for biomarkers IL-8, total protein, albumin, sRAGE, PAF-AcH, and IL-6. Since most of the included studies were case-control studies, we excluded studies with other design types. Heterogeneity for total protein, albumin, and IL-6 was partly explained. However, heterogeneity for IL-8 was not clarified. Only one article was case-control study; therefore, the source of heterogeneity was not determined for PAF-AcH because of a limited number of study. We also excluded studies with ARDS that was not diagnosed using AECC criteria, which significantly reduced the heterogeneity for IL-6, but not for albumin. In addition, we assumed sample type may be a variable between studies because biomarker measurement in BALF was influenced by recovery rate and dilution. IL-8 remained significantly increased when BALF studies were excluded. As only one article measured biomarkers in lung fluid other than BALF, it was not evaluated. Results of the subgroup analysis are presented in Additional file 6.

### Biomarkers associated with ARDS mortality

**Fig. 3:**
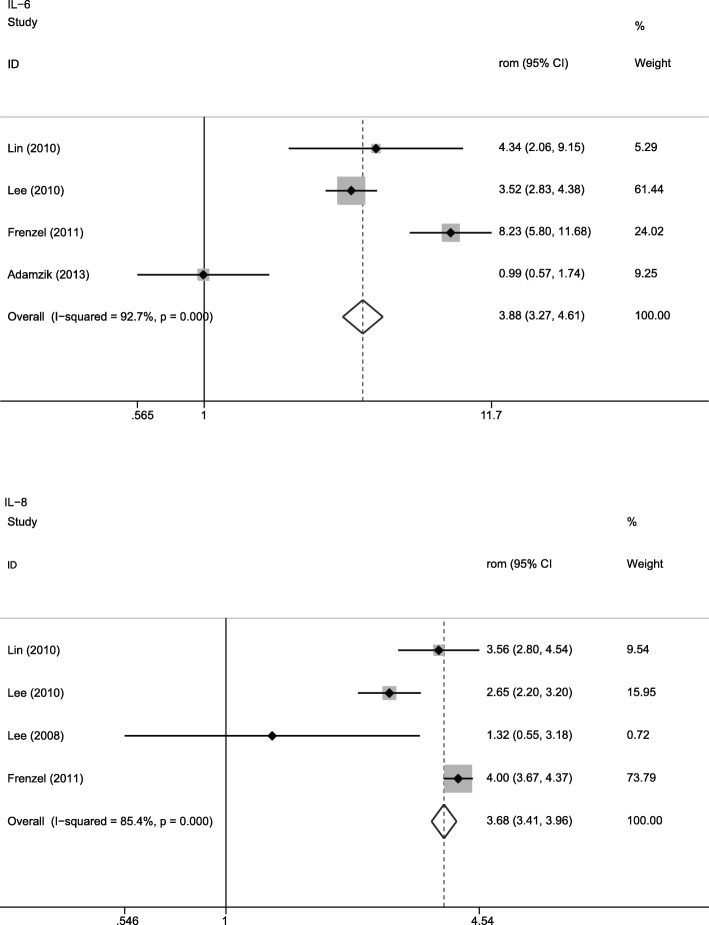
Forest plot for acute respiratory distress syndrome (ARDS) mortality. RoM, ratio of means; CI, confident interval; IL-6, interleukin-6; IL-8, interleukin-8

We performed a meta-analysis on 11 biomarkers in lung fluid associated with ARDS mortality (Table 3); Fig. 3 shows the forest plots for biomarkers associated with ARDS mortality. Interleukin-1β (IL-1β) (4.617 [4.331, 4.921]), IL-6 (3.882 [3.270, 4.608]), IL-8 (3.679 [3.414, 3.964]), and Kerbs von Lungren-6 (KL-6) (3.178 [2.931, 3.446]) ranked the highest in biomarkers associated with ARDS mortality. The overall effect size ranged from 0.406 to 4.617. Among the biomarkers with a significant difference between survivors and non-survivors, decreased levels of interleukin-2 (IL-2) (0.828 [0.715, 0.959]) and CC16 (0.406 [0.362, 0.405]) were associated with a high mortality rate. Heterogeneity was displayed for many of the biomarkers, and influence analysis indicated that the heterogeneities were not likely caused by extreme RoM values. Due to the small number of studies, subgroup analysis based on design type or sample type could not be performed. Subgroup analysis for tumor necrosis factor-α (TNF-α) was performed when excluding patients with ARDS not diagnosed using AECC criteria. Results of the subgroup analysis are presented in Additional file 7.

**Table 3:** Biomarkers associated with ARDS mortality

Biomarker	No. of study	No. of patients	RoM (95% CI)	*p*	Heterogeneity	
					*Q* (*p* value)	*I*^2^ (%)
Interleukin-1β	3[12, 24, 39]	137	4.617 (4.331, 4.921)	< 0.05	11.24 (0.004)	82.20
Interleukin-6	4[12, 24, 39, 41]	176	3.882 (3.270, 4.608)	< 0.05	41.09 (< 0.1)	92.70
Interleukin-8	4[24, 38, 39, 41]	160	3.679 (3.414, 3.964)	< 0.05	20.59 (< 0.1)	85.40
Kerbs von Lungren-6	2[30, 34]	65	3.178 (2.931, 3.446)	< 0.05	0.83 (0.363)	0.00
Plasminogen activator inhibitor-1	2[52, 56]	32	2.085 (2.039, 2.133)	< 0.05	1.31 (0.252)	23.70
Tumor necrosis factor-α	3[12, 24, 39]	137	1.923 (1.656, 2.233)	< 0.05	11.47 (0.003)	82.60
Procollagen peptide III	3[17, 19, 42]	194	1.714 (1.613, 1.822)	< 0.05	34.08 (< 0.1)	94.10
Total protein	3[12, 19, 42]	208	1.667 (1.595, 1.742)	< 0.05	82.47 (< 0.1)	97.60
Interleukin-2	2[13, 40]	27	0.828 (0.715, 0.959)	0.012	1.49 (0.223)	32.70
Club cell protein	2[31, 35]	37	0.406 (0.362, 0.405)	< 0.05	21.08 (< 0.1)	95.30
Interleukin-10	3[12, 24, 39]	137	1.019 (0.922, 1.127)	0.709	54.54 (< 0.1)	96.30

Numbers within the square brackets were reference numbers

*RoM* ratio of means, *CI* confident interval

### Publication bias

Among the biomarkers associated with ARDS, Egger’s regression test demonstrated a *p* value of less than 0.10 for IL-6; furthermore, when we adjusted for possible publication bias by Duval and Tweedie’s trim and fill, the RoM remained significant for IL-6. Among the biomarkers associated with mortality, no publication bias was noted. The results of the publication bias analysis are presented in Additional file 8.

## Discussion

In this systematic review and meta-analysis, we summarized the biomarkers related to ARDS diagnosis in the at-risk population and those related to ARDS mortality.

By searching several databases and screening for related articles, 49 studies involving 2189 patients were identified.

We discovered that total protein, albumin, PAI-1, PAF-ACH, sTNFα-RII, HGF, IL-8, PCP I, PCP III, soluble receptor for advanced glycation end products (SRAGE), and IL-6 were significantly increased in the lung fluid of patients with ARDS. Although total PLA2 activity, soluble intercellular adhesion molecule-1 (SICAM-1), IL-2, CC16, and MMP-9 were also significantly different between patients with ARDS and at-risk patients, few studies were included on each of these biomarkers, so the results are unreliable.

IL-1β, IL-6, IL-8, TNF-α, PCPIII, and total protein were significantly increased in lung fluid of patients who died in the ARDS cohort. Furthermore, few studies for KL-6, plasminogen activator inhibitor-1 (PAI-1), IL-2, and CC16 were included, although these biomarkers were significantly different between survivors and non-survivors.

Pioneering work by Terpstra et al. reported on plasma biomarkers for ARDS diagnosis and prognosis. They reported that KL-6, lactate dehydrogenase (LDH), SRAGE, von Willebrand factor (vWF), and IL-8 displayed the highest effect size for ARDS diagnosis, and interleukin-4 (IL-4), IL-2, Angiopoietin-2 (Ang-2), and KL-6 had the highest effect size for ARDS prognosis (assessed by pooled odds ratio). These biomarkers represent pathophysiological processes, which led to the hypothesis that ARDS diagnosis is correlated with tissue damage, whereas ARDS mortality is correlated with systemic inflammation (4). In our meta-analysis, biomarkers for ARDS diagnosis were related to inflammation (IL-8 and IL-6), endothelial injury (SICAM), epithelial injury (SRAGE and HGF), lung fibroproliferation (PCPI and PCPIII), and coagulopathy (PAF-ACH). With regard to ARDS mortality, biomarkers related to inflammation (IL-8, IL-6, and IL-1β), epithelial injury (KL-6), and lung fibroproliferation (PCPIII) were elevated in the lung fluid of patients with ARDS presenting the worst outcomes. Therefore, we assume that, aside from tissue damage and systemic inflammation, lung fibroproliferation is vital in both ARDS diagnosis and prognosis. Several studies on lung biopsy of patients with ARDS showed a strong relationship between fibrosis activity and ARDS mortality [64, 65].

To our knowledge, this is the first meta-analysis of biomarkers in lung fluid for ARDS. Since lower respiratory tract specimens are now easily obtained, BALF and other lung fluids have become frequently used clinical samples for pulmonary disease diagnosis, secondary to plasma/serum. Since the most intensive physiological processes in ARDS occur in the lung, theoretically, lung fluid can reflect the pathophysiological process differently from other body fluids.

In this analysis, we applied RoM to assess the effect size and to attempt to eliminate the bias caused by dilution of different kinds of lung fluid. For example, edema fluid was completely undiluted, whereas BALF might be quite diluted. This methodology has been proved to be robust and is widely used [7–9, 66, 67].

This systematic review and meta-analysis may prompt further research on ARDS diagnosis and prognosis in many different ways. First, it demonstrates the research priorities and indicates that research on ARDS biomarkers in lung fluid and other compartments is needed. Second, it establishes ARDS biomarkers and their performance in lung fluid as an innovative research field. Third, it identifies numerous novel translational approaches for biomarker measurement in different compartments, such as chromatography for metabolomics separation and mass spectrometry or nuclear magnetic resonance spectroscopy for biomarker detection [68]. Although some non-quantitative methods were not included in this meta-analysis, they are still worth exploring.

There were limitations in this meta-analysis as well. First, although we performed a subgroup analysis of the biomarkers related to ARDS diagnosis and mortality, heterogeneity was not explainable for every biomarker. We assume that this could be related to the different etiologies of ARDS, variation in BALF procedures between studies, multiple control types used in the studies, wide range of intervals between study inclusion and biomarker measurement, and different treatments for ARDS. We were not able to conduct further analysis due to the limited information.

Second, a limited number of studies were included for each biomarker, which impedes the reproducibility of the results. The number of studies for each biomarker should be taken into consideration while assessing the performance in the ranking system.

Third, only the biomarkers addressed by two or more studies were included. Because of this, promising biomarkers evaluated in a single study were not considered, which may limit the view on lung fluid biomarker research as a whole.

Finally, the use of lung fluid as a study object itself had some limitations. Due to the lack of information on specific BALF procedures, we could only assume the recovery rates between subgroups in one study were within an acceptable range, which may have caused some of the heterogeneity between studies.

## Conclusions

This systematic review and meta-analysis included 49 studies with 2189 participants, providing an overview of research on lung fluid biomarkers for ARDS. The ranking system provided by evaluating the effect size for ARDS diagnosis and prognosis may serve as a reference for further research on biomarkers for ARDS.

## Additional files

Additional file 1: Search strategy. (DOCX 17 kb)

Additional file 2: Tailored QUADAS-2. (DOCX 17 kb)

Additional file 3: ARDS etiologies. (DOCX 13 kb)

Additional file 4: Result of quality assessment. Low = low possibility of risk of bias. High = means high possibility of high risk of bias. Index test(s) = measurement used in an article for biomarker concentration detection. Reference standard = criteria used for acute respiratory distress syndrome diagnosis, including American European Consensus Conference criteria, lung injury score, Fowler criteria and so on. Flow and timing is a part evaluating possible bias of the process including patients recruiting and biomarker measurement. (EPS 2305 kb)

Additional file 5: Outcome of influence analysis. (DOCX 14 kb)

Additional file 6: Result of subgroup analysis for diagnosis. (DOCX 14 kb)

Additional file 7: Result of subgroup analysis for mortality. (DOCX 12 kb)

Additional file 8: Result of publication bias. (DOCX 13 kb)

## Abbreviations

AECC: American European Consensus Conference
ALI: Acute lung injury
Ang-2: Angiopoietin-2
ARDS: Acute respiratory distress syndrome
BALF: Bronchoalveolar lavage fluid
CC16: Club cell protein
ELF: Epithelial lining fluid
IL-1β: Interleukin-1β
IL-2: Interleukin-2
IL-4: Interleukin-4
IL-8: Interleukin-8
KL-6: Kerbs von Lungren-6
LAF: Lung aspirational fluid
LDH: Lactate dehydrogenase
MMP-9: Matrix metalloproteinases-9
OR: Odds ratio
PAF-ACH: Platelet activating factor-acetyl choline
PAI-1: Plasminogen activator inhibitor-1
PCP I: Procollagen peptide I
PCP III: Procollagen peptide III
PEF: Pulmonary edema fluid
PLA2: Phospholipases A2
QUADAS-2: Quality Assessment of Diagnostic Accuracy Studies Score-2
RoM: Ratio of means
SD: Standard deviation
SE: Standard error
SICAM: Soluble intercellular adhesion molecule-1
SRAGE: Soluble receptor for advanced glycation end products
STNF-RII: Soluble TNF-α receptors II
TNF-α: Tumor necrosis factor-α
vWF: von Willebrand factor
