# Supplementary material for: Lung fluid biomarkers for acute respiratory distress syndrome: a systematic review and meta-analysis
Source: Crit Care. 2019 Feb 12;23:43. doi: 10.1186/s13054-019-2336-6 (PMC6373030; doi:10.1186/s13054-019-2336-6)
Supplement: Supplementary file 1 — Search strategy. (DOCX 17 kb) [file 13054_2019_2336_MOESM1_ESM.docx]

**Supplemental Digital Content 1**

1. **Search strategy for PubMed**

((((((((((((inflammation[MeSH Terms]) OR inflammation*) OR inflammatory)) OR (((((("endothelial cells"[MeSH Terms]) OR "Cell*, Endothelial") OR "Endothelial Cell*") OR "Cell*, Vascular Endothelial") OR "Vascular Endothelial Cell*") OR "Capillary Endothelial Cell*")) OR ((((((((("alveolar epithelial cells"[MeSH Terms]) OR "Alveolar Epithelial Cell*") OR "Pneumocyte*") OR "Alveolar Cell*") OR "Cell*, Alveolar") OR "Type-II Pneumocyte*") OR "Type 2 Pneumocyte*") OR "Type-I Pneumocyte*") OR "Type 1 Pneumocyte*"))) AND (("Biomarkers"[Mesh]) OR (((((((((((((((((“Markers*, Biological”) OR “Biologic Marker*”) OR “Biological Marker*”) OR “Laboratory Marker*”) OR “Marker*, Laboratory”) OR “Marker*, Serum”) OR “Serum Marker*”) OR “Surrogate End Point*”) OR “Surrogate Endpoint*”) OR “Clinical Marker*”) OR “Viral Marker*”) OR “Biochemical Marker*”) OR “Marker*, Biochemical”) OR “Immune Marker*”) OR “Immunologic Marker*”) OR “Surrogate Marker*”) OR "biomarker*"))) AND ((((("Acute Lung Injur*"[MeSH Terms]) OR “Lung Injur*, Acute”[Title/Abstract]) OR ALI[Title/Abstract])) OR (("Respiratory Distress Syndrome, Adult"[Mesh]) OR ((((((“Shock Lung”[Title/Abstract]) OR “Human ARDS”[Title/Abstract]) OR “Respiratory Distress Syndrome, Acute”[Title/Abstract]) OR “Acute Respiratory Distress Syndrome”[Title/Abstract]) OR “Adult Respiratory Distress Syndrome”[Title/Abstract]) OR ARDS[Title/Abstract])))) AND (((((((("Bronchoalveolar Lavage Fluid"[MeSH Terms]) OR (((((("Bronchial Alveolar Lavage Fluid*"[Title/Abstract]) OR "Pulmonary Lavage Fluid*"[Title/Abstract]) OR "Lavage Fluid*, Lung"[Title/Abstract]) OR "Lung Lavage Fluid*"[Title/Abstract]) OR "Alveolar Lavage Fluid*"[Title/Abstract]) OR "Bronchial Lavage Fluid*"[Title/Abstract]) OR "BAL-F") OR "BAL fluid*") OR ((“Edema fluid*”) AND (("Lung"[Mesh]) OR ((lung[Title/Abstract]) OR pulmonary[Title/Abstract])))) OR “Lung edema fluid*”) OR “Pulmonary edema fluid*”) OR “BALF”))) OR (((((((("Bronchoalveolar Lavage Fluid"[MeSH Terms]) OR (((((("Bronchial Alveolar Lavage Fluid*"[Title/Abstract]) OR "Pulmonary Lavage Fluid*"[Title/Abstract]) OR "Lavage Fluid*, Lung"[Title/Abstract]) OR "Lung Lavage Fluid*"[Title/Abstract]) OR "Alveolar Lavage Fluid*"[Title/Abstract]) OR "Bronchial Lavage Fluid*"[Title/Abstract]) OR "BAL-F") OR "BAL fluid*") OR ((“Edema fluid*”) AND (("Lung"[Mesh]) OR ((lung[Title/Abstract]) OR pulmonary[Title/Abstract])))) OR “Lung edema fluid*”) OR “Pulmonary edema fluid*”) OR “BALF”))) AND ((((("Acute Lung Injur*"[MeSH Terms]) OR “Lung Injur*, Acute”[Title/Abstract]) OR ALI[Title/Abstract])) OR (("Respiratory Distress Syndrome, Adult"[Mesh]) OR ((((((“Shock Lung”[Title/Abstract]) OR “Human ARDS”[Title/Abstract]) OR “Respiratory Distress Syndrome, Acute”[Title/Abstract]) OR “Acute Respiratory Distress Syndrome”[Title/Abstract]) OR “Adult Respiratory Distress Syndrome”[Title/Abstract]) OR ARDS[Title/Abstract])))) AND (((((((("Matrix Metalloproteinases"[Mesh]) OR ((("Matrix Metalloproteinase") OR MMP) OR MMPs))) OR (("Elastin"[Mesh]) OR (((((("Elastin") OR "Elastins") OR "kappa-Elastin") OR "kappa Elastin") OR "alpha-Elastin") OR "alpha Elastin"))) OR (("Laminin"[Mesh]) OR (((((((Laminin) OR laminins) OR "Glycoprotein GP-2") OR "Glycoprotein GP 2") OR Merosin) OR "Laminin M") OR "Laminin M Chain"))) OR ((Fibrocytes) OR Fibrocyte)) OR ((("Myofibroblasts"[MeSH Terms]) OR Myofibroblasts) OR Myofibroblast)) OR ((((("procollagen"[MeSH Terms]) OR Procollagen) OR "Procollagen peptides") OR "Procollagen Type M") OR Protocollagen)))) OR (((((((("Bronchoalveolar Lavage Fluid"[MeSH Terms]) OR (((((("Bronchial Alveolar Lavage Fluid*"[Title/Abstract]) OR "Pulmonary Lavage Fluid*"[Title/Abstract]) OR "Lavage Fluid*, Lung"[Title/Abstract]) OR "Lung Lavage Fluid*"[Title/Abstract]) OR "Alveolar Lavage Fluid*"[Title/Abstract]) OR "Bronchial Lavage Fluid*"[Title/Abstract]) OR "BAL-F") OR "BAL fluid*") OR ((“Edema fluid*”) AND (("Lung"[Mesh]) OR ((lung[Title/Abstract]) OR pulmonary[Title/Abstract])))) OR “Lung edema fluid*”) OR “Pulmonary edema fluid*”) OR “BALF”))) AND ((((("Acute Lung Injur*"[MeSH Terms]) OR “Lung Injur*, Acute”[Title/Abstract]) OR ALI[Title/Abstract])) OR (("Respiratory Distress Syndrome, Adult"[Mesh]) OR ((((((“Shock Lung”[Title/Abstract]) OR “Human ARDS”[Title/Abstract]) OR “Respiratory Distress Syndrome, Acute”[Title/Abstract]) OR “Acute Respiratory Distress Syndrome”[Title/Abstract]) OR “Adult Respiratory Distress Syndrome”[Title/Abstract]) OR ARDS[Title/Abstract])))) AND (("Biomarkers"[Mesh]) OR (((((((((((((((((“Markers*, Biological”) OR “Biologic Marker*”) OR “Biological Marker*”) OR “Laboratory Marker*”) OR “Marker*, Laboratory”) OR “Marker*, Serum”) OR “Serum Marker*”) OR “Surrogate End Point*”) OR “Surrogate Endpoint*”) OR “Clinical Marker*”) OR “Viral Marker*”) OR “Biochemical Marker*”) OR “Marker*, Biochemical”) OR “Immune Marker*”) OR “Immunologic Marker*”) OR “Surrogate Marker*”) OR "biomarker*")))

1. **Search strategy for Embase**

((('acute respiratory distress syndrome':ab,ti OR ('adult respiratory distress syndrome'/exp OR 'adult respiratory distress syndrome') OR 'adult respiratory distress':ab,ti OR ards:ab,ti OR 'lung shock':ab,ti OR 'posttraumatic lung failure':ab,ti OR 'posttraumatic pulmonary insufficiency':ab,ti OR 'respiratory distress syndrome, acute':ab,ti OR 'respiratory distress syndrome, adult':ab,ti OR 'respiratory distress, adult':ab,ti OR 'shock lung':ab,ti) OR (('acute lung injury'/exp OR 'acute lung injury') OR ('lipopolysaccharide-induced acute lung injury'/exp OR 'lipopolysaccharide-induced acute lung injury') OR ('transfusion related acute lung injury'/exp OR 'transfusion related acute lung injury') OR 'acute lung injuries':ab,ti OR ali:ab,ti OR 'lung injury, acute':ab,ti OR 'lung injuries, acute':ab,ti OR 'lipopolysaccharide-induced acute pulmonary injury':ab,ti OR 'lps-induced acute lung injury':ab,ti OR trali:ab,ti)) AND (('biological marker'/exp OR 'biological marker') OR 'indicator, biological' OR 'marker, biological' OR bioindicator OR 'biological indicator' OR 'biological markers' OR biomarker OR biomarkers) AND (('bronchoalveolar lavage fluid'/exp OR 'bronchoalveolar lavage fluid') OR 'broncho-alveolar lavage fluid' OR 'broncho-alveolar washings' OR 'bronchoalveolar washings' OR 'pulmonary edema fluid' OR 'lung edema fluid' OR 'edema fluid,lung' OR 'bal fluid' OR balf)) OR ((('acute respiratory distress syndrome':ab,ti OR ('adult respiratory distress syndrome'/exp OR 'adult respiratory distress syndrome') OR 'adult respiratory distress':ab,ti OR ards:ab,ti OR 'lung shock':ab,ti OR 'posttraumatic lung failure':ab,ti OR 'posttraumatic pulmonary insufficiency':ab,ti OR 'respiratory distress syndrome, acute':ab,ti OR 'respiratory distress syndrome, adult':ab,ti OR 'respiratory distress, adult':ab,ti OR 'shock lung':ab,ti) OR (('acute lung injury'/exp OR 'acute lung injury') OR ('lipopolysaccharide-induced acute lung injury'/exp OR 'lipopolysaccharide-induced acute lung injury') OR ('transfusion related acute lung injury'/exp OR 'transfusion related acute lung injury') OR 'acute lung injuries':ab,ti OR ali:ab,ti OR 'lung injury, acute':ab,ti OR 'lung injuries, acute':ab,ti OR 'lipopolysaccharide-induced acute pulmonary injury':ab,ti OR 'lps-induced acute lung injury':ab,ti OR trali:ab,ti)) AND (('biological marker'/exp OR 'biological marker') OR 'indicator, biological' OR 'marker, biological' OR bioindicator OR 'biological indicator' OR 'biological markers' OR biomarker OR biomarkers) AND (('bronchoalveolar lavage fluid'/exp OR 'bronchoalveolar lavage fluid') OR 'broncho-alveolar lavage fluid' OR 'broncho-alveolar washings' OR 'bronchoalveolar washings' OR 'pulmonary edema fluid' OR 'lung edema fluid' OR 'edema fluid,lung' OR 'bal fluid' OR balf) AND (('inflammation'/exp OR 'inflammation') OR 'acute inflammation' OR 'inflammation reaction' OR 'inflammation response' OR 'inflammatory condition' OR 'inflammatory lesion' OR 'inflammatory process' OR 'inflammatory reaction' OR 'inflammatory response' OR 'inflammatory syndrome' OR 'reaction, inflammation' OR 'response, inflammatory')) OR ((('acute respiratory distress syndrome':ab,ti OR ('adult respiratory distress syndrome'/exp OR 'adult respiratory distress syndrome') OR 'adult respiratory distress':ab,ti OR ards:ab,ti OR 'lung shock':ab,ti OR 'posttraumatic lung failure':ab,ti OR 'posttraumatic pulmonary insufficiency':ab,ti OR 'respiratory distress syndrome, acute':ab,ti OR 'respiratory distress syndrome, adult':ab,ti OR 'respiratory distress, adult':ab,ti OR 'shock lung':ab,ti) OR (('acute lung injury'/exp OR 'acute lung injury') OR ('lipopolysaccharide-induced acute lung injury'/exp OR 'lipopolysaccharide-induced acute lung injury') OR ('transfusion related acute lung injury'/exp OR 'transfusion related acute lung injury') OR 'acute lung injuries':ab,ti OR ali:ab,ti OR 'lung injury, acute':ab,ti OR 'lung injuries, acute':ab,ti OR 'lipopolysaccharide-induced acute pulmonary injury':ab,ti OR 'lps-induced acute lung injury':ab,ti OR trali:ab,ti)) AND (('biological marker'/exp OR 'biological marker') OR 'indicator, biological' OR 'marker, biological' OR bioindicator OR 'biological indicator' OR 'biological markers' OR biomarker OR biomarkers) AND (('bronchoalveolar lavage fluid'/exp OR 'bronchoalveolar lavage fluid') OR 'broncho-alveolar lavage fluid' OR 'broncho-alveolar washings' OR 'bronchoalveolar washings' OR 'pulmonary edema fluid' OR 'lung edema fluid' OR 'edema fluid,lung' OR 'bal fluid' OR balf) AND (('endothelium cell'/exp OR 'endothelium cell') OR 'cell, endothelium' OR 'endothelial cell' OR 'endothelial cells' OR 'endothelial lining cell' OR 'littoral cell')) OR ((('acute respiratory distress syndrome':ab,ti OR ('adult respiratory distress syndrome'/exp OR 'adult respiratory distress syndrome') OR 'adult respiratory distress':ab,ti OR ards:ab,ti OR 'lung shock':ab,ti OR 'posttraumatic lung failure':ab,ti OR 'posttraumatic pulmonary insufficiency':ab,ti OR 'respiratory distress syndrome, acute':ab,ti OR 'respiratory distress syndrome, adult':ab,ti OR 'respiratory distress, adult':ab,ti OR 'shock lung':ab,ti) OR (('acute lung injury'/exp OR 'acute lung injury') OR ('lipopolysaccharide-induced acute lung injury'/exp OR 'lipopolysaccharide-induced acute lung injury') OR ('transfusion related acute lung injury'/exp OR 'transfusion related acute lung injury') OR 'acute lung injuries':ab,ti OR ali:ab,ti OR 'lung injury, acute':ab,ti OR 'lung injuries, acute':ab,ti OR 'lipopolysaccharide-induced acute pulmonary injury':ab,ti OR 'lps-induced acute lung injury':ab,ti OR trali:ab,ti)) AND (('biological marker'/exp OR 'biological marker') OR 'indicator, biological' OR 'marker, biological' OR bioindicator OR 'biological indicator' OR 'biological markers' OR biomarker OR biomarkers) AND (('bronchoalveolar lavage fluid'/exp OR 'bronchoalveolar lavage fluid') OR 'broncho-alveolar lavage fluid' OR 'broncho-alveolar washings' OR 'bronchoalveolar washings' OR 'pulmonary edema fluid' OR 'lung edema fluid' OR 'edema fluid,lung' OR 'bal fluid' OR balf) AND (('lung alveolus epithelium cell'/exp OR 'lung alveolus epithelium cell') OR 'alveolar epithelial cell' OR 'alveolar epithelial cells' OR 'alveolar epithelium cell' OR 'alveolar epithelium cells' OR 'epithelial alveolar cell' OR 'epithelial alveolar cells' OR 'epithelium alveolar cell' OR 'epithelium alveolar cells' OR 'lung alveolar epithelial cell' OR 'lung alveolar epithelial cells')) OR ((('acute respiratory distress syndrome':ab,ti OR ('adult respiratory distress syndrome'/exp OR 'adult respiratory distress syndrome') OR 'adult respiratory distress':ab,ti OR ards:ab,ti OR 'lung shock':ab,ti OR 'posttraumatic lung failure':ab,ti OR 'posttraumatic pulmonary insufficiency':ab,ti OR 'respiratory distress syndrome, acute':ab,ti OR 'respiratory distress syndrome, adult':ab,ti OR 'respiratory distress, adult':ab,ti OR 'shock lung':ab,ti) OR (('acute lung injury'/exp OR 'acute lung injury') OR ('lipopolysaccharide-induced acute lung injury'/exp OR 'lipopolysaccharide-induced acute lung injury') OR ('transfusion related acute lung injury'/exp OR 'transfusion related acute lung injury') OR 'acute lung injuries':ab,ti OR ali:ab,ti OR 'lung injury, acute':ab,ti OR 'lung injuries, acute':ab,ti OR 'lipopolysaccharide-induced acute pulmonary injury':ab,ti OR 'lps-induced acute lung injury':ab,ti OR trali:ab,ti)) AND (('biological marker'/exp OR 'biological marker') OR 'indicator, biological' OR 'marker, biological' OR bioindicator OR 'biological indicator' OR 'biological markers' OR biomarker OR biomarkers) AND (('bronchoalveolar lavage fluid'/exp OR 'bronchoalveolar lavage fluid') OR 'broncho-alveolar lavage fluid' OR 'broncho-alveolar washings' OR 'bronchoalveolar washings' OR 'pulmonary edema fluid' OR 'lung edema fluid' OR 'edema fluid,lung' OR 'bal fluid' OR balf) AND (('fibrogenesis'/exp OR 'fibrogenesis') OR 'fibrogenicity')) OR ((('acute respiratory distress syndrome':ab,ti OR ('adult respiratory distress syndrome'/exp OR 'adult respiratory distress syndrome') OR 'adult respiratory distress':ab,ti OR ards:ab,ti OR 'lung shock':ab,ti OR 'posttraumatic lung failure':ab,ti OR 'posttraumatic pulmonary insufficiency':ab,ti OR 'respiratory distress syndrome, acute':ab,ti OR 'respiratory distress syndrome, adult':ab,ti OR 'respiratory distress, adult':ab,ti OR 'shock lung':ab,ti) OR (('acute lung injury'/exp OR 'acute lung injury') OR ('lipopolysaccharide-induced acute lung injury'/exp OR 'lipopolysaccharide-induced acute lung injury') OR ('transfusion related acute lung injury'/exp OR 'transfusion related acute lung injury') OR 'acute lung injuries':ab,ti OR ali:ab,ti OR 'lung injury, acute':ab,ti OR 'lung injuries, acute':ab,ti OR 'lipopolysaccharide-induced acute pulmonary injury':ab,ti OR 'lps-induced acute lung injury':ab,ti OR trali:ab,ti)) AND (('bronchoalveolar lavage fluid'/exp OR 'bronchoalveolar lavage fluid') OR 'broncho-alveolar lavage fluid' OR 'broncho-alveolar washings' OR 'bronchoalveolar washings' OR 'pulmonary edema fluid' OR 'lung edema fluid' OR 'edema fluid,lung' OR 'bal fluid' OR balf) AND (('procollagen'/exp OR 'procollagen') OR 'collagen precursor' OR 'precollagen' OR 'protocollagen' OR ('myofibroblast'/exp OR 'myofibroblast') OR myofibroblasts OR ('fibrocyte'/exp OR 'fibrocyte') OR fibrocytes OR ('laminin'/exp OR 'laminin') OR laminine OR ('elastin'/exp OR 'elastin') OR 'elastin like protein' OR ('matrix metalloproteinase'/exp OR 'matrix metalloproteinase') OR 'matrix metallopeptidase' OR 'matrix metalloproteinases' OR 'matrix metalloproteinases, secreted' OR matrixin))

1. **Search strategy for Wed of Science**

TOPIC: ((“adult respiratory distress syndrome” OR “acute respiratory distress syndrome” OR ards OR “lung shock” OR “shock lung” OR “respiratory distress syndrome, acute” OR “respiratory distress syndrome, adult”) OR TOPIC: (“acute Lung Injur*” OR ali)) AND TOPIC: (biomarker* OR biological marker* OR bioindicator* OR biological indicator*) AND TOPIC: (“bronchoalveolar lavage fluid*” OR “bronchial alveolar lavage fluid*” OR “pulmonary lavage fluid*” OR “lung lavage fluid*” OR “alveolar lavage fluid*” OR “bronchial lavage fluid” OR “pulmonary edema fluid*” OR “lung edema fluid*” OR bal OR “bal fluid*” OR balf)

1. **Search strategy for Cochrane Library**

**#1** MeSH descriptor: [Respiratory Distress Syndrome, Adult] explode all trees

**#2** "Respiratory Distress Syndrome, Acute"

**#3** "Acute Respiratory Distress Syndrome"

**#4**“Adult Respiratory Distress Syndrome”

**#5** ARDS

**#6** #1 or #2 or #3 or #4 or #5

**#7** MeSH descriptor: [Acute Lung Injury] explode all trees

**#8** "Acute Lung Injur*"

**#9** "Lung Injur*, Acute"

**#10** "Transfusion Related Acute Lung Injury"

**#11** #7 or #8 or #9 or #10

**#12** #6 or #11

**#13** MeSH descriptor: [Bronchoalveolar Lavage Fluid] explode all trees

**#14** "Pulmonary Lavage Fluid*"

**#15** "Lung Lavage Fluid*"

**#16** "Alveolar Lavage Fluid*"

**#17** "Bronchial Lavage Fluid*"

**#18** BALF

**#19** "BAL fluid*"

**#20** "Pulmonary edema fluid*"

**#21** "Edema fluid*"

**#22** #14 or #15 or #16 or #17 or #18 or #19 or #20 or #21

**#23** MeSH descriptor: [Biomarkers] explode all trees

**#24** "Marker*, Biological"

**#25** "Biologic Marker*"

**#26** "Biological Marker*"

**#27** "Marker*, Laboratory"

**#28** "Laboratory Marker*"

**#29** "Marker*, Clinical"

**#30** "Clinical Marker*"

**#31** "Biochemical Marker*"

**#32** "Marker*, Biochemical"

**#33** "Marker*, Immunologic"

**#34** "Immune Marker*"

**#35** "Marker*, Immune"

**#36** "Immunologic Marker*"

**#37** "Surrogate Marker*"

**#38** "Marker*, Surrogate"

**#39** #23 or #24 or #25 or #26 or #27 or #28 or #29 or #30 or #31 or #32 or#33 or #34 or #35 or #36 or #37 or #38

**#40** #12 and #22 and #39
