# Supplementary material for: Lung fluid biomarkers for acute respiratory distress syndrome: a systematic review and meta-analysis
Source: Crit Care. 2019 Feb 12;23:43. doi: 10.1186/s13054-019-2336-6 (PMC6373030; doi:10.1186/s13054-019-2336-6)
Supplement: Supplementary file 2 — Tailored QUADAS-2. (DOCX 17 kb) [file 13054_2019_2336_MOESM2_ESM.docx]

**Supplemental Digital Content 2**

The Quality Assessment of Diagnostic Accuracy Studies-2 (QUADAS-2) is a tool developed for systematic reviews of diagnostic accuracy studies transformed from the original QUADAS tool, it’s comprised of 4 parts: patient selection, index test, reference standard, and flow and timing. With the risk of bias and applicability concerns graph and summary, visualized judgement of quality can be obtained.

However, because of the diversity of diagnostic systematic reviews, questions have to be tailored to fit in each study by adding or omitting questions.

Since most of our studies included were case-control studies, Signaling question 2 in domain 1 (*Was a case–control design avoided?*) was removed and replaced by “*Were there clear inclusion and exclusion criteria?* Signaling question 2 in domain 2: “*If a threshold was used, was it prespecified?”* were left out since no threshold was taken into consideration in our meta-analysis.

The exact QUADAS-2 tool used in this meta-analysis was listed below.

**I PATIENT SELECTION**

**Risk of bias**

Were there clear inclusion and exclusion criteria?

Yes / No / Unclear

Was a consecutive or random sample of patients enrolled?

Yes / No / Unclear

Did the study avoid inappropriate exclusion?

Yes / No / Unclear

Could the selection of patients have introduced bias?

Low / High / Unclear

**Applicability**

Is there concern that the included patients do not match the review question?

Low / High / Unclear

**II INDEX TEST(S)**

**Risk of bias**

Were the index test results interpreted without knowledge of the results of the reference standard?

Yes / No / Unclear

Could the conduct or interpretation of the index test have introduced bias?

Low / High / Unclear

**Applicability**

Is there concern that the index test, its conduct, or interpretation differ from the review question?

Low / High / Unclear

**III REFERENCE STANDARD**

**Risk of bias**

Is the reference standard likely to correctly classify the target condition?

Yes / No / Unclear

Were the reference standard results interpreted without knowledge of the result of the index test?

Yes / No / Unclear

Could the reference standard, its conduct, or its interpretation have introduced bias?

Low / High / Unclear

**Applicability**

Is there concern that the target condition as defined by the reference standard does not match the review question?

Low / High / Unclear

**IV flow and timing**

**Risk of bias**

Was there an appropriate interval between index test(s) and reference standard?

Yes / No / Unclear

Did all patients receive a reference standard?

Yes / No / Unclear

Did patients receive the same reference standard?

Yes / No / Unclear

Were all patients included in the analysis?

Yes / No / Unclear

Could the patients flow have introduced bias？

Low / High / Unclear

**Annotations:** **“Low”** implies low possibility of risk of bias, **“High”** means high possibility of high risk of bias. **“Index test(s)”** indicates the measurement used in an article for biomarker concentration detection. **“Reference standard”** in this meta-analysis implies to criteria used for acute respiratory distress syndrome diagnosis, including American European Consensus Conference criteria, lung injury score, Fowler criteria and so on. **“Flow and timing”** is a part evaluating possible bias of the process including patients recruiting and biomarker measurement.
