# Supplementary material for: Lung fluid biomarkers for acute respiratory distress syndrome: a systematic review and meta-analysis
Source: Crit Care. 2019 Feb 12;23:43. doi: 10.1186/s13054-019-2336-6 (PMC6373030; doi:10.1186/s13054-019-2336-6)
Supplement: Supplementary file 3 — ARDS etiologies. (DOCX 13 kb) [file 13054_2019_2336_MOESM3_ESM.docx]

Table1 Etiology of ARDS

| Etiology of ARDS | N (%) |
| --- | --- |
| Sepsis | 409(30.87) |
| Pneumonia | 314(23.70) |
| Trauma | 145(10.94) |
| Aspiration | 113(8.53) |
| Transfusion | 56(4.23) |
| Major surgery | 46(3.47) |
| not provided | 162(12.23) |
| Other | 80(6.04) |

ARDS=Acute Respiratory Distress Syndrome
