# Supplementary material for: Lung fluid biomarkers for acute respiratory distress syndrome: a systematic review and meta-analysis
Source: Crit Care. 2019 Feb 12;23:43. doi: 10.1186/s13054-019-2336-6 (PMC6373030; doi:10.1186/s13054-019-2336-6)
Supplement: Supplementary file 5 — Outcome of influence analysis. (DOCX 14 kb) [file 13054_2019_2336_MOESM5_ESM.docx]

Table2 Influence analysis

|  | |  |  |  |  |  | corrected heterogeneity | |
| --- | --- | --- | --- | --- | --- | --- | --- | --- |
| Biomarker | Removed studies | | No. of patients removed | Remaining  studies | No. of patients remaining | Corrected RoM(95%CI) | I²% | P |
| Total protein | | 2 | 106 | 3 | 73 | 9.391(6.866,12.845) | 0 | 0.404 |
| Albumin | | 1 | 37 | 4 | 154 | 1.962 (1.136,3.388) | 0 | 0.683 |
| Platelet Activating Factor-acetyl choline | | 2 | 58 | 2 | 62 | 4.046 (2.718,6.024) | 7.3 | 0.299 |
| Interleukin-8 | | 2 | 234 | 5 | 143 | 3.985 (2.762,5.751) | 0 | 0.908 |
| Interleukin-6 | | 1 | 112 | 4 | 138 | 3.833 (1.958,7.502) | 0 | 0.865 |

No. = Number, RoM=Ratio of means, CI=Confident Interval
