# Supplementary material for: Lung fluid biomarkers for acute respiratory distress syndrome: a systematic review and meta-analysis
Source: Crit Care. 2019 Feb 12;23:43. doi: 10.1186/s13054-019-2336-6 (PMC6373030; doi:10.1186/s13054-019-2336-6)
Supplement: Supplementary file 6 — Result of subgroup analysis for diagnosis. (DOCX 14 kb) [file 13054_2019_2336_MOESM6_ESM.docx]

Table3 Subgroup analysis for biomarkers associated with diagnosis

|  |  |  |  | Heterogeneity | | |
| --- | --- | --- | --- | --- | --- | --- |
|  | No. of studies | RoM (95%CI) | P value | Q(P value) | I²% |  |
| **Design type**  Total protein | 2 | 6.949(3.999，12.077) | ＜0.05 | 0.13(0.72) | 0 |  |
| Albumin | 2 | 0.76(-1.28,2.80) | 0.057 | 15(0) | 93.3 |  |
| Interleukin-8 | 5 | 5.734(4.098,8.022) | ＜0.05 | 11.26(0.024) | 64.5 |  |
| Interleukin-6 | 3 | 3.551(1.741,7.236) | ＜0.05 | 0.33(0.848) | 0 |  |
| **Diagnositic criteria** |  |  |  |  |  |  |
| Interleukin-6 | 2 | 4.442(1.373,14.369) | 0.013 | 0.31(0.576) | 0 |  |
| **Sample type** |  |  |  |  |  |  |
| Interleukin-8 | 3 | 4.219(2.641,6.738) | ＜0.05 | 0.12(0.941) | 0 |  |

No. =Number, RoM=Ratio of means, CI=Confident Interval
