# Supplementary material for: Lung fluid biomarkers for acute respiratory distress syndrome: a systematic review and meta-analysis
Source: Crit Care. 2019 Feb 12;23:43. doi: 10.1186/s13054-019-2336-6 (PMC6373030; doi:10.1186/s13054-019-2336-6)
Supplement: Supplementary file 7 — Result of subgroup analysis for mortality. (DOCX 12 kb) [file 13054_2019_2336_MOESM7_ESM.docx]

Table4 Subgroup analysis for biomarkers associated with mortality

|  |  |  |  | Heterogeneity | | |
| --- | --- | --- | --- | --- | --- | --- |
|  | No. of studies | RoM (95%CI) | P value | Q(P value) | I²% |  |
| **Diagnositic criteria** |  |  |  |  |  |  |
| Tumor Necrosis Factor-α | 2 | 1.452(1.158,1.819) | 0.001 | 0.86(0.355) | 0 |  |

No. =Number, RoM=Ratio of means, CI=Confident Interval
