# Supplementary material for: Lung fluid biomarkers for acute respiratory distress syndrome: a systematic review and meta-analysis
Source: Crit Care. 2019 Feb 12;23:43. doi: 10.1186/s13054-019-2336-6 (PMC6373030; doi:10.1186/s13054-019-2336-6)
Supplement: Supplementary file 8 — Result of publication bias. (DOCX 13 kb) [file 13054_2019_2336_MOESM8_ESM.docx]

Table5 Publication bias

|  | Egger's regression | | Duval & Tweedie’s trim and fil | | |
| --- | --- | --- | --- | --- | --- |
| Biomarkers | Z | P value | Original RoM(95%CI) | studies trimmed | Adjusted RoM(95%CI) |
| Interleukin-6 | 1.47 | 0.032 | 1.826（1.170,2.852） | 3 | 1.423（0.948，2.136） |

RoM=Ratio of means, CI= Confident Interval
